# Solubility enhancement of aggregation-prone heterologous proteins by fusion expression using stress-responsive *Escherichia coli *protein, RpoS

**DOI:** 10.1186/1472-6750-8-15

**Published:** 2008-02-19

**Authors:** Jin-Seung Park, Kyung-Yeon Han, Jong-Ho Lee, Jong-Am Song, Keum-Young Ahn, Hyuk-Seong Seo, Sang-Jun Jun Sim, Seung-Wook Kim, Jeewon Lee

**Affiliations:** 1 Department of Chemical and Biological Engineering, Korea University, Anam-Dong 5-1, Sungbuk-Ku, Seoul 136-713, South Korea; 2 Department of Chemical Engineering, Sungkyunkwan University, Suwon, South Korea

## Abstract

**Background:**

The most efficient method for enhancing solubility of recombinant proteins appears to use the fusion expression partners. Although commercial fusion partners including maltose binding protein and glutathione-*S*-transferase have shown good performance in enhancing the solubility, they cannot be used for the proprietory production of commercially value-added proteins and likely cannot serve as universal helpers to solve all protein solubility and folding issues. Thus, novel fusion partners will continue to be developed through systematic investigations including proteome mining presented in this study.

**Results:**

We analyzed the *Escherichia coli *proteome response to the exogenous stress of guanidine hydrochloride using 2-dimensional gel electrophoresis and found that RpoS (RNA polymerase sigma factor) was significantly stress responsive. While under the stress condition the total number of soluble proteins decreased by about 7 %, but a 6-fold increase in the level of RpoS was observed, indicating that RpoS is a stress-induced protein. As an N-terminus fusion expression partner, RpoS increased significantly the solubility of many aggregation-prone heterologous proteins in *E. coli *cytoplasm, indicating that RpoS is a very effective solubility enhancer for the synthesis of many recombinant proteins. RpoS was also well suited for the production of a biologically active fusion mutant of *Pseudomonas putida *cutinase.

**Conclusion:**

RpoS is highly effective as a strong solubility enhancer for aggregation-prone heterologous proteins when it is used as a fusion expression partner in an *E. coli *expression system. The results of these findings may, therefore, be useful in the production of other biologically active industrial enzymes, as successfully demonstrated by cutinase.

## Background

*Escherichia coli *have been widely used as a host to produce valuable commercial, industrial, and therapeutic proteins. There are several disadvantages with this system, however, especially for the expression of eukaryotic proteins. For example, when randomly selected 2,078 full-length genes of *Caenorhabditis elegans *were expressed in *E. coli *cytoplasm, only 11% of genes yielded significant amounts of soluble material [1]. Several different approaches have been taken to resolve the solubility problem in the past and include (1) truncation of long multi-domain proteins into short and separate domains [2] ; (2) co-expression of molecular chaperones or foldases [3]; (3) enabled secretion to the periplasm where disulfide bonds can be properly formed with the help of an oxidative environment and Dsb protein families [4]; (4) co-expression of aminoacyl tRNA cognates to amino acids encoded by rare codons [5]; and more recently (5) use of a fusion expression partner [6,7]. The most efficient method for enhancing solubility and folding efficiencies of recombinant proteins appears to be the latter (fusion expression partners) which includes maltose binding protein (MBP) [8], thioredoxin (Trx) [9], human ferritin heavy chains (hFTN-H) [10], and glutathione-*S*-transferase (GST) [11]. Although these fusion partners have shown good performance in enhancing the solubility and folding of some recombinant proteins [12], they likely cannot serve as universal helpers to solve all protein solubility and folding issues. Thus, novel fusion partners will continue to be developed through systematic investigations including proteome mining.

In the present study, we found that the level of RpoS significantly increased during the stress caused by guanidine hydrochloride through proteome-wide mining involving the stress response of *E. coli *BL21(DE3). As an N-terminus fusion expression partner, RpoS dramatically increased the solubility of the following heterologous proteins (human minipro-insulin (mp-INS), human epidermal growth factor (EGF), human prepro-ghrelin (ppGRN), human interleukin-2 (hIL-2), human activation induced cytidine deaminase (AID), human glutamate decarboxylase (GAD448–585), *Pseudomonas putida *cutinase (CUT), human ferritin light chain (hFTN-L), human granulocyte colony-stimulating factor (G-CSF), and cold autoinflammatory syndrome1 (NALP3) NACHT domain (NACHT)). We also have demonstrated an increased yield of a biologically active fusion mutant of heterologous bacterial cutinase that is expected to be of significant biotechnical and commercial interest.

## Results and discussion

### Guanidine hydrochloride-induced proteome response of *E. coli *and finding of the aggregation-resistant protein, RpoS

**Figure 1:**
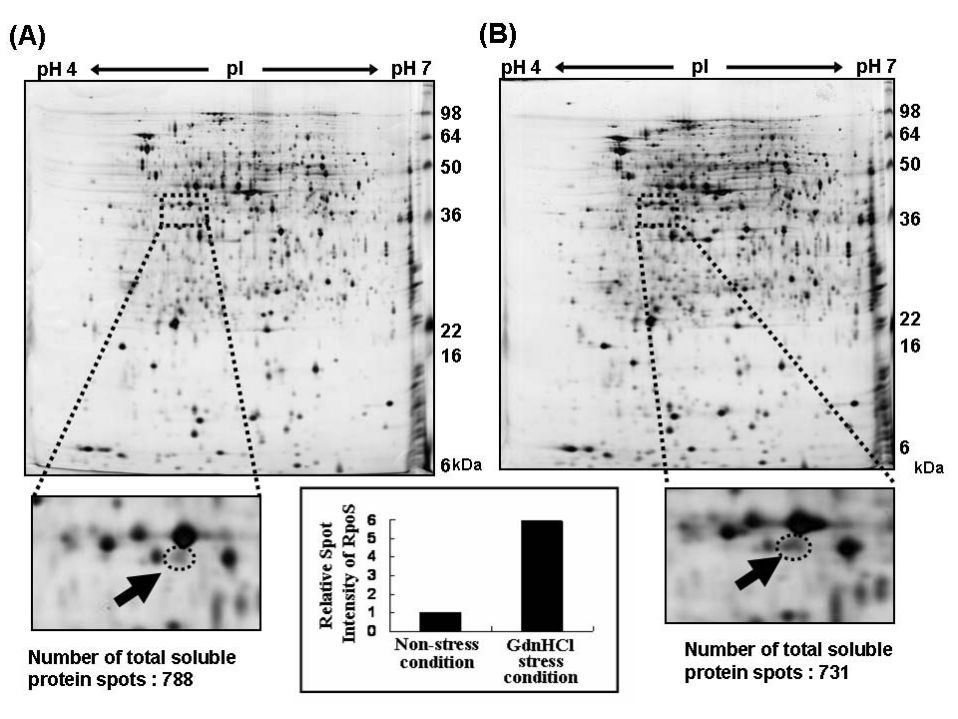
***E. coli *proteome profiles under non-stress and GdnHCl-stress condition**. (A) 2-DE gel image of *E. coli *proteome under the non-stress condition. (Arrow indicates RpoS spot under non-stress condition.) (B) 2-DE gel image of *E. coli *proteome under GdnHCl-stress condition (Arrow indicates RpoS spot under GdnHCl-stress condition). (Figure in a box presents relative spot intensities of RpoS, analyzed under non-stress and GdnHCl-stress conditions.)

Exogenous stresses including heat shock and GdnHCl [13-15] often results in the misfolding and aggregation of proteins within the cell. Therefore, it would seem reasonable to presume that intracellular proteins which exist in their native soluble forms even under stress conditions have an intrinsic ability to remain efficiently folded compared to proteins that unfold or aggregate under the same stresses. GdnHCl stress facilitates the release of periplasmic proteins through increased membrane permeability [16-18] and hence could be regarded as an osmolyte. We investigated changes of the proteome profile of *E. coli *when the stress reagent, guanidine hydrochloride, was added to growing bacterial cultures, by applying 2-dimensional gel electrophoresis (2-DE). Compared to non-stress condition, the total number of intracellular soluble proteins was reduced to 731 from 788, which indicates that many proteins aggregated under the stress condition induced by GdnHCl. What was surprising is that the RpoS expression level (i.e. protein spot intensity estimated through analysis of 2-DE gel images, Fig. 1. and Table 1) increased 6-fold compared to the non-stress situation. In addition to RpoS, we found several other proteins (Table 1), the expression level of which more than 1.9-fold increased in response to the stressor GdnHCl, including HSP60 chaperonin (57 kD), chaperone HtpG (71 kD), transcription elongation protein NusA (55 kD), DNA gyrase subunit A (97 kD), formate acetyltransferase 1 (85 kD), succinate dehydrogenase flavoprotein subunit (64 kD), *etc*. Since the synthesis yield of target protein can be significantly reduced, the molecular mass of proteins to be used as fusion expression partner should not be too high. That is, although the large amount of fusion protein is synthesized, the actual amount of the target fusion-free protein could be very low if the fusion partner is too big. RpoS (38 kD) is a relatively small protein among the stress-responsive proteins we found and was highly effective in enhancing the solubility of target proteins.

RpoS, stress responsive RNA polymerase sigma factor is a well-known universal stress regulator controlling many proteins under various stress conditions, e.g. the onset of the stationary phase [19], and carbon starvation [20]. RpoS was previously reported to be induced by osmotic stress [16] and heat shock [21]. We also observed that the RpoS expression level increased 3- to 5-fold in response to heat shock (data not shown). Muffler *et al*. [21] reported that the duration of stability of RpoS in response to heat shock stress is maintained by the direct- or indirect-binding of DnaK. Bound DnaK appears to assist the effective folding of RpoS and also protect RpoS against the action of ClpP, a protease that degrades RpoS [21]. From this point of view, RpoS may serve as a solubility enhancer in *E. coli *cytoplasm, when used as a fusion expression partner upon the expression of aggregation-prone heterologous proteins.

**Table 1:** Result of proteome-wide finding of some aggregation-resistant *E. coli *proteins

**Gene name^a^**	**Access No.^a^**	**Protein name^a^**	**pI/MW(kDa)**		**Sequence coverage^d^**	**Score^d^**	**Fold change**
						
			**Theoretical^b^**	**Experimental^c^**			
rpoS	P13445	RNA polymerase sigma factor rpoS	4.89/37.97	5.08/38.95	19	36	6.21
groL	P0A6F5	HSP60 chaperonin	4.85/57.20	4.90/58.63	16	34	2.13
htpG	P0A6Z3	Chaperone protein htpG	5.09/71.38	5.14/66.37	9	27	3.10
nusA	P0AFF6	Transcription elongationprotein nusA	4.53/54.87	4.69/59.09	16	44	3.46
gyrA	P0AES4	DNA gyrase subunit A	5.09/96.96	5.20/92.23	21	29	1.95
pflB	P09373	Formate acetyltransferase 1	5.69/85.23	5.68/83.51	19	39	3.26
sdhA	P0AC41	Succinate dehydrogenase flavoprotein subunit	5.85/64.42	5.70/65.73	25	43	2.16

^a ^Gene name, accession number, and protein name were obtained from ExPASy Proteomics Server [35].

^b ^Theoretical values of pI and Mw were calculated using "Compute pI/Mw tool" [36].

^c ^Experimental values of pI and Mw were estimated through 2-DE gel image analysis in the present study.

^d ^Sequence coverage and score values were calculated using "ALDENTE : PEPTIDE MASS FINGERPRINTING TOOL" [37].

### Expression of aggregation-prone heterologous proteins using RpoS as fusion partner

**Figure 2:**
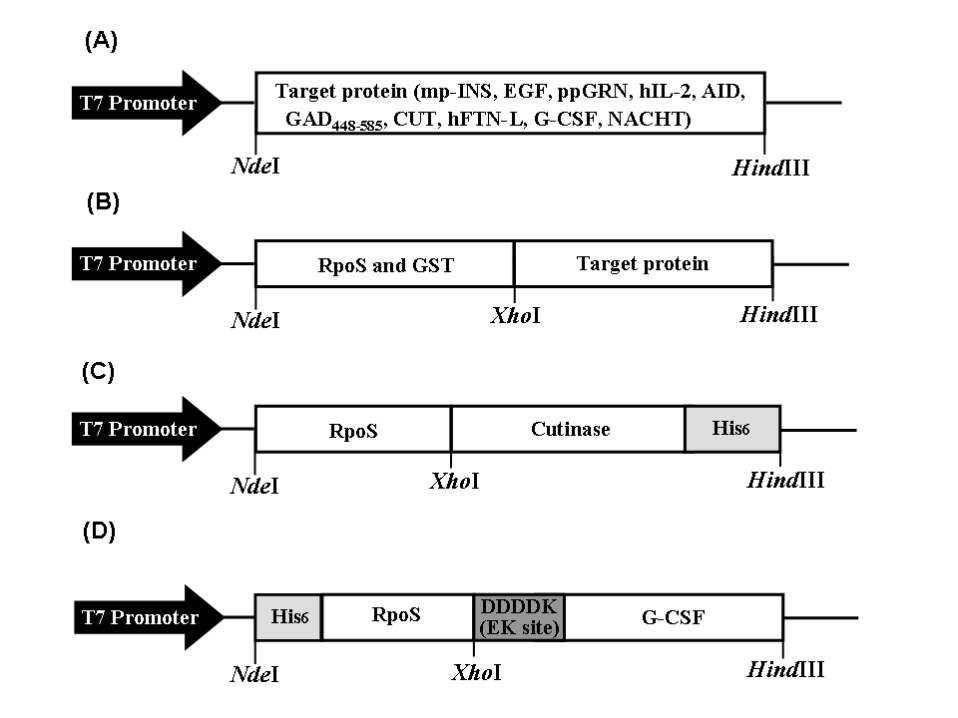
**The plasmid vector constructions for direct and fusion expression of heterologous proteins in *E. coli***. (A) Direct expression vector, (B) RpoS- and GST-fusion expression vector, (C) Hybrid vector for metal (Ni^+2^) affinity purification of RpoS-CUT-(His)_6_, (D) Hybrid vector for metal (Ni^+2^) affinity purification of (His)_6_-RpoS-D_4_K-G-CSF, followed by enterokinase cleavage.

**Figure 3:**
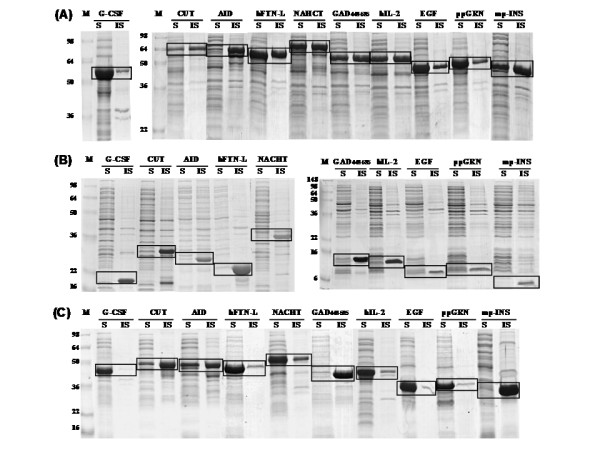
**Results of direct and fusion expression of heterologous proteins**. SDS-PAGE analyses of the RpoS-fusion expressed proteins (A), directly (non-fusion) expressed proteins (B), and GST-fusion expressed proteins (C).

We used RpoS as an N-terminus fusion expression partner for the synthesis of numerous heterologous proteins [mp-INS, EGF, ppGRN, hIL-2, AID, GAD_448–585_, CUT, hFTN-L, G-CSF, and NACHT (Fig. 2 and Table 2 for expression system construction)]. Stenström *et al*. [22] reported that the codon following an AUG start triplet (+2 codon) significantly affects gene expression in *E. coli*, and the second codon starting with A is most advantageous to achieve an enhanced expression level. The second codon of RpoS is AGT (encoding Ser) that seems to be favorable second codon based on the report of Stenström *et al*. [22]. As shown in Figure 3B and Table 3, all heterologous proteins expressed directly without RpoS fusion formed insoluble inclusion bodies resulting in nearly negligible solubility. Compared to these results, it seems surprising that when the same heterologous proteins were expressed with the fusion of RpoS, the solubility of these foreign proteins dramatically increased (Fig. 3A and Table 3), thereby indicating that *E. coli *RpoS is a highly effective solubility enhancer for aggregation-prone heterologous proteins. Table 3 compares the effect of RpoS- and GST fusion on the solubility enhancement for heterologous proteins. In the fusion expression of CUT, GAD_448–585_, and mpINS, the effect of RpoS fusion was much higher, whereas GST fusion was significantly more effective in the expression of AID, NACHT, and hFTN-L. This result indicates that there are no universal helpers to solve all protein solubility issues. Table 3 also shows that the results of direct- and fusion expression of heterologous proteins are highly reproducible.

**Table 2:** Primers used for the cloning of genes encoding various heterologous proteins

**Heterologous proteins**	**Primer sequences**		
		**Direct expression**	**Fusion expression**
mp-INS	Sense	**cat atg **ttt gtc aac caa cat	**ctc gag **ttt gtc aac caa cat
	Antisense	**aag ctt **tta gtt aca gta gtt c	**aag ctt **tta gtt aca gta gtt c
EGF	Sense	**cat atg **aac tct gac tcc gaa tgc	**ctc gag **aac tct gac tcc gaa tgc
	Antisense	**aag ctt **tta acg cag ttc cca cca	**aag ctt **tta acg cag ttc cca cca
ppGRN	Sense	**cat atg **ggc tcc agc ttc ctg	**ctc gag **ggc tcc agc ttc ctg
	Antisense	**aag ctt **tca ctt gtc ggc t	**aag ctt **tca ctt gtc ggc t
hIL-2	Sense	**cat atg **gca cct act tca agt	**ctc gag **gca cct act tca agt
	Antisense	**aag ctt **tta tca agt cag tgt	**aag ctt **tta tca agt cag tgt
AID	Sense	**cat atg **gac agc ctc ttg atg aac	**ctc gag **gac agc ctc ttg atg aac
	Antisense	**aag ctt **tca taa caa aag tcc ca	**aag ctt **tca taa caa aag tcc ca
GAD_448–585_	Sense	**cat atg **cgc cac gtt gat gt	**ctc gag **cgc cac gtt gat gt
	Antisense	**atc gat **tta taa atc ttg tcc	**atc gat **tta taa atc ttg tcc
CUT	Sense	**cat atg **gct ccc ctg ccg gat ac	**ctc gag **gct ccc ctg ccg gat ac
	Antisense	**aag ctt **tta aag ccc gcg gcg ct	**aag ctt **tta aag ccc gcg gcg ct
			**aag ctt **atg atg gtg gtg atg atg tta aag ccc gcg gcg ct (for metal affinity purification)
hFTN-L	Sense	**cat atg **agc tcc cag att cgt	**ctc gag **agc tcc cag att cgt
	Antisense	**aag ctt **tta gtc gtg ctt gag agt	**aag ctt **tta gtc gtg ctt gag agt
G-CSF	Sense	**cat atg **act cca ctc gga cct g	**ctc gag **acc ccc ctg ggc cct gcc
			**ctc gag **gac gat gac gat aaa acc ccc ctg ggc cct gcc (for enterokinase digestion)
	Antisense	**aag ctt **tca tgg ctg tgc aag	**aag ctt **tca tgg ctg tgc aag
NACHT	Sense	**cat atg **act gtg gtg ttc cag	**ctc gag **act gtg gtg ttc cag
	Antisense	**aag ctt **tca cag cag gta gta c	**aag ctt **tca cag cag gta gta c

**Table 3:** Solubility of the expressed recombinant proteins

**Heterologous proteins**	Solubility* of the expressed recombinant proteins (%)		
	
	RpoS-fusion expression	GST-fusion expression	Direct expression
G-CSF	87.4 ± 2.1	91.5 ± 1.5	4.6 ± 0.5
CUT	44.9 ± 2.7	26.2 ± 1.1	3.1 ± 0.2
AID	18.1 ± 3.5	41.7 ± 1.5	8.7 ± 1.6
hFTN-L	47.8 ± 2.1	87.6 ± 2.8	1.7 ± 0.4
NACHT	36.4 ± 4.3	74.8 ± 2.2	8.4 ± 1.3
GAD_448–585_	59.0 ± 1.8	7.8 ± 2.7	1.9 ± 0.5
hIL-2	60.1 ± 2.4	85.7 ± 1.7	1.3 ± 0.3
EGF	80.4 ± 0.9	92.9 ± 1.2	3.0 ± 0.9
ppGRN	89.2 ± 3.5	90.1 ± 2.3	7.6 ± 0.6
mp-INS	75.9 ± 2.8	13.3 ± 1.0	1.3 ± 0.4

* The solubility was defined as the fraction of the soluble recombinant protein compared to the synthesized total (soluble + insoluble) recombinant protein. Average and standard deviation values were calculated based on the results of repeated triplicate experiments.

**Figure 4:**
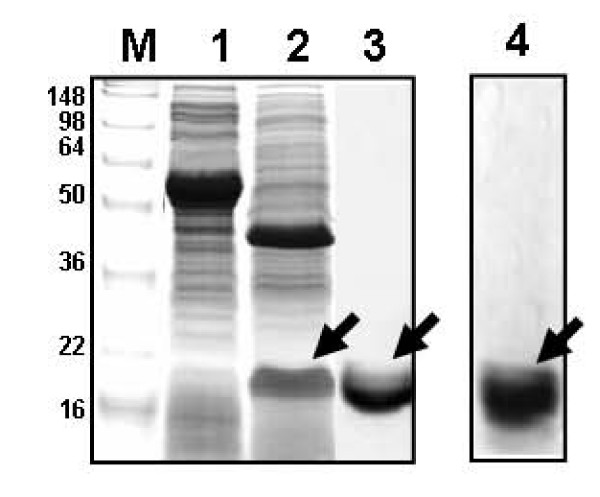
**Results of SDS-PAGE and Western blot analysis of RpoS-fusion and fusion-free G-CSF**. -SDS-PAGE analysis (lane M-3): lane M, molecular markers; lane 1, supernatant of recombinant *E. coli *cell lysates containing recombinant (His)_6_-RpoS-D_4_K-G-CSF, which was loaded onto ProBond resin (Ni^+2^) column for metal affinity purification; lane 2, soluble fraction of enterokinase(EK)-digested product of (His)_6_-RpoS-D_4_K-G-CSF, containing RpoS and fusion-free G-CSF (indicated by an arrow); lane 3, purified soluble fusion-free G-SCF. -Western blot analysis (lane 4): result of immunoblotting analysis of purified soluble fusion-free G-SCF (loaded onto lane 3).

Moreover, the plasmid vector for the expression of polyhistidine-tagged fusion mutant of G-CSF [(His)_6_::RpoS::(D_4_K)::G-CSF] was constructed (Fig. 2) to purify fusion-free G-CSF. After (His)_6_::RpoS::(D_4_K)::G-CSF was bound onto the ProBond resin (Ni^+2^) column, the enterokinase proteolysis was carried out in a batch mode, and subsequently the digested product was collected and centrifuged. SDS-PAGE and Western blot analyses of the supernatant show that the recombinant G-CSF was easily released from the Rpos-fusion protein and was present in the form of soluble protein in the supernatant (Fig. 4).

### Bioactivity of the recombinant fusion mutant of cutinase, RpoS::CUT

Cutinase has been used as a lipolytic enzyme in the composition of laundry and dishwashing detergents to more efficiently remove immobilized fats [23,24]. In addition, the oleochemistry industries [25], and pollutant degradation [26,27] represent other potential uses of cutinase. In recent years, the esterification and transesterification properties of cutinase have been intensively exploited and could be applied usefully in other chemical synthesis processes [28]. Because of these extensive potential applications, we were particularly interested in the production of a bioactive recombinant cutinase. Thus, we cloned the cutinase gene from the genome of *Pseudomonas putida *and expressed it in *E. coli *using the fusion of RpoS. Cutinase is known for its hydrolytic activity for a variety of esters ranging from soluble p-nitrophenyl esters to insoluble long-chain triglycerides. The hydrolytic activity of cutinase, especially on *p*-nitrophenyl esters of fatty acids, is extremely sensitive to fatty acid chain length. Previously, Lin *et al*. [29] reported that microbial cutinase lacked a large hydrophobic surface around the active site, in contrast to other lipases and esterases. This structural characteristic of cutinase may be strongly related to high substrate-specificity, i.e. the extremely low activity on *p*-nitrophenyl ester of a long chain fatty acid such as *p*-nitrophenyl palmitate (PNP).

**Figure 5:**
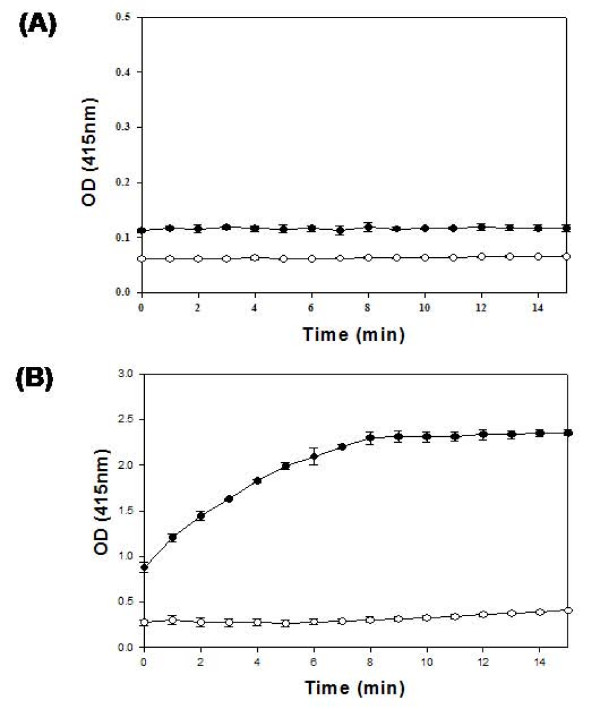
**Bioactivity of recombinant fusion mutant, RpoS::CUT**. Assay results using cell-free supernatants from (A) *E. coli *BL21 (DE3) host and (B) recombinant *E. coli *BL21 (DE3) [pT7-RpoS-CUT] producing RpoS::CUT. Both PNB (●) and PNP (○) were used as substrates for the cutinase activity assay. (Concentrations: PNB = 6.6 mM; PNP = 6.6 mM).

**Figure 6:**
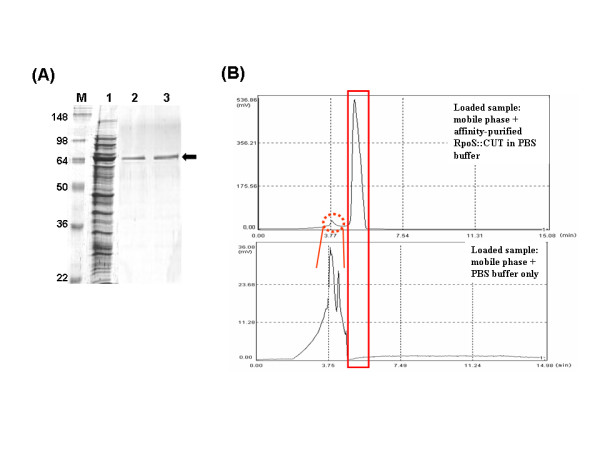
**Results of purified RpoS::CUT-6xHis analysis**. (A) Results of SDS-PAGE analysis of RpoS::CUT-6xHis in soluble fraction of *E. coli *cell lysates (lane 1) and of purified RpoS::CUT-6xHis (lanes 2, 3). (M: molecular marker) (Arrow indicates recombinant RpoS::CUT-6xHis). (B) Result of reversed-phase HPLC analysis of the affinity-purified RpoS::CUT-6xHis. (Peaks in red rectangle and dotted circle correspond to the purified RpoS::CUT-6xHis and PBS buffer, respectively.)

We assayed the enzymatic activity of our fusion mutant of cutinase (RpoS::CUT) and demonstrated the same selective bioactivity as native cutinase to degrade *p*-nitrophenyl butyrate (PNB) but not to degrade PNP (Fig. 5A and 5B). We also purified RpoS::CUT-His_6 _(Fig. 6A) through Ni^2+ ^affinity chromatography and analyzed the purified RpoS::CUT-His_6 _using reversed phase HPLC (Fig. 6B). As shown in Figure 6B, RpoS::CUT-His_6 _was analyzed as a single peak, which seems to indicate that the crafted mutant molecules of cutinase have uniform and correctly folded conformation due probably to the help of fusion partner, *E. coli *RpoS.

## Conclusion

Using 2-dimensional gel electrophoresis, we found that *E. coli *RpoS, RNA polymerase sigma factor was GdnHCl stress-responsive and highly effective as a strong solubility enhancer when used as fusion partner for the expression of aggregation-prone heterologous proteins in *E. coli *BL21(DE3). The results of these findings may, therefore, be useful in the production of other biologically active industrial enzymes, as successfully demonstrated by cutinase.

## Methods

### Bacterial strain and plasmids

*E. coli *strain BL21(DE3) (F^-^*omp*T *hsd*S_B_(rB^- ^mB^-^)) was selected under both non-stress and GdnHCl-stress conditions for 2-dimensional gel electrophoresis analysis. Through PCR amplification using appropriate primers (Table 2), the genes encoding mp-INS, EGF, ppGRN, hIL-2 [30], AID, GAD_448–585 _[31], CUT, hFTN-L [32], G-CSF, and NACHT were cloned using previously cloned heterologous genes, except for CUT gene that was cloned from chromosomal DNA of *Pseudomonas putida *(ATCC 53552). The gene clones of mp-INS, ppGRN, AID, G-CSF, and NACHT were kindly donated by other researchers, as acknowledged (see Acknowledgements). The EGF gene was cloned using pCMV6-XL vector (OriGene Technologies, USA). Each of the recombinant genes above and various fusion/hybrid genes [*rpo*S(or GST gene)::(each heterologous gene)] were inserted into the *Nde*I-*Hin*dIII site of the same plasmid pT7-7 to construct the expression vectors. All the heterologous genes above were fused directly to the RpoS gene (cloned from the chromosomal DNA of *E. coli *BL21(DE3)) or GST gene [cloned from the GST-fusion vector pET42a(+) (Novagen, USA)] without any linker sequence. Therefore, each expression vector has no enzymatic cleavage site between *rpo*S (or GST gene) and heterologous gene, except for the case of G-CSF. For the purification of fusion-free recombinant G-CSF, the D_4_K sequence for enterokinase digestion was inserted between RpoS and G-CSF genes to synthesize the polyhistidine-tagged fusion mutant of G-CSF, i.e. (His)_6_::RpoS::(D_4_K)::G-CSF. For the purification of RpoS::CUT, hexahistidine (His_6_) was added to the C-terminus of CUT by PCR (Fig. 2). For this, the sequence coding for *N-*RpoS::CUT-His_6_*-C *was inserted into the *Nde*I-*Hin*dIII site of plasmid pT7-7.

After complete DNA sequencing of all gel-purified hybrid plasmids, the *E. coli *strain BL21(DE3) (F^- ^*omp*T *hsd*S_B_(rB^- ^mB^-^)) was transformed with the hybrid plasmids, and ampicillin-resistant transformants were subsequently selected using LB-agar plates supplemented with ampicillin (100 mg/l).

### Recombinant *E. coli *culture, gene expression, and recombinant protein purification

For shake flask experiments, 250 ml Erlenmeyer flasks containing 50 ml LB media and ampicillin at 100 mg/l of culture (37°C and 150 rpm) was used. When the culture turbidity (OD_600_) reached 0.5, gene expression was induced with the addition of IPTG (1 mM), and after a further 4 h of cultivation the all recombinant cells (80 mg wet cell mass) were harvested by centrifugation (13,000 rpm (MICRO17TR, Hanil Science Industrial, Korea) × 5 min) and cell pellets resuspended in 5 ml lysis buffer (10 mM Tris-HCl, pH 7.5, 10 mM EDTA). Cell disruption was achieved using a Branson Sonifier (Branson Ultrasonics Corp., Danbury, CT). The cell-free supernatant and insoluble protein aggregates were separated at 13,000 rpm (MICRO17TR, Hanil Science Industrial, Korea) for 10 min. The isolated inclusion bodies, if any, were washed twice with 1 % Triton X-100. Cell-free supernatants and the washed inclusion bodies were subjected to polyacrylamide (14%) gel electrophoresis (PAGE) analysis. Coomassie-stained protein bands were ultimately scanned, and the intensity of each recombinant protein band was estimated using densitometry (Duoscan T1200, Bio-Rad, Hercules, CA). The solubility of recombinant proteins was determined by analyzing the fraction of the soluble recombinant protein compared to the synthesized total (soluble + insoluble) recombinant protein. Average and standard deviation values of the solubility were calculated based on the results of repeated triplicate experiments.

The purification of recombinant G-SCF was accomplished using metal affinity chromatography. That is, polyhistidine-tagged fusion mutant of G-CSF [(His)_6_::RpoS::(D_4_K)::G-CSF] (Fig. 4) were loaded onto ProBond resin (Ni^+2^) column. Prior to sample loading, the resin was washed twice with 10 column volumes of binding buffer (50 mM potassium phosphate, 300 mM KCl, 20 mM imidazole, pH 7.0). Binding buffer contains 20 mM imidazole to minimize non-specific binding of untagged protein contaminants, and binding was carried out in a batch mode at 4°C. Afterwards the resin was washed twice with 5–8 ml Tris-HCl (10 mM Tris, pH 8.0) prior to enterokinase digestion step. The enterokinase digestion was carried out in a batch mode at 4°C for 10 h using 5-unit enterokinase (Invitrogen, CA, USA). Then, the proteolytic product was collected and centrifuged [3,000 rpm (MICRO17TR, Hanil Science Industrial, Korea) for 10 min], and the supernatant fraction was subjected to being analyzed by SDS-PAGE and western blotting.

### Purification and HPLC analysis of cutinase fusion mutant

For the purification of RpoS::CUT-His_6_, the soluble fraction was separated after cell disruption by centrifugation (13,000 rpm (MICRO17TR, Hanil Science Industrial, Korea)) for 30 min. The cell-free supernatant containing RpoS::CUT-His_6 _was loaded onto the ProBond resin (Ni^2+^) column (Invitrogen) for affinity purification. Before the sample loading, the resin was washed twice with ten column volumes of binding buffer (pH 8.0, 50 mM sodium phosphate, 300 mM NaCl, 10 mM imidazole). Binding was carried out in a batch mode at 4°C. Afterwards, the resin was washed twice with 8 ml washing buffer (pH 8.0, 50 mM sodium phosphate, 300 mM NaCl, 50 mM imidazole) and eluted with elution buffer (pH 8.0, 50 mM sodium phosphate, 300 mM NaCl, 250 mM imidazole). The elution buffer in the eluted solution containing the purified RpoS::CUT-His_6 _was changed with PBS buffer (137 mM NaCl, 2.7 mM KCl, 10 mM Na_2_HPO_4_, 2 mM KH_2_PO_4_, pH 7.4) using Amicon Ultra-4 centrifugal filter (Millipore, Ireland). Analysis of the purified RpoS::CUT-His_6 _was performed with high-performance liquid chromatography (HPLC) (LC-20A Prominence, Shimadzu Co. Ltd., Japan). A Shim-pack CLC-NH2 column (60 × 150 mm, Shimadzu Co. Ltd., Japan) was equilibrated with 10 % acetonitrile containing 0.1 % trifluoroacetic acid at a flow rate of 1 ml/min. The sample was loaded onto the column and eluted with 75 % acetonitrile. The elution profile was monitored at 215 nm.

### Sample preparation for proteome analysis and 2-dimensioanl gel electrophoresis

Flask culture conditions were identical to those for recombinant gene expressions. Cells were grown at 37°C and then 100 mM GdnHCl was added for the GdnHCl-induced proteome response when culture turbidity (OD_600_) reached 0.5 in the LB media. After further 3 h cultivation, the cells were harvested by centrifugation at 6,000 rpm (MEGA17R, Hanil Science Industrial, Korea)for 15 min (4°C) and then washed twice with 40 mM Tris buffer (pH 8.0). Cell pellets were resuspended in 500 μl of lysis buffer (8 M urea, 4% (w/v) CHAPS, 40 mM Tris, protease inhibitor cocktail; Roche Diagnostics GmbH, Mannheim, Germany) and disrupted by sonication. After sonication (Branson Sonifier 450, USA), the cell debris and the aggregated proteins were removed by centrifugation at 12,000 rpm (MICRO17TR, Hanil Science Industrial, Korea) for 60 min (4°C), and only soluble proteins were obtained. 2-dimensional gel electrophoresis was performed as described previously Kim *et al*. [33]. Stained gels were scanned using a UMAX powerlook 1100 scanner and Image Master software v 4.01 (Amersham Biosciences, Uppsala, Sweden) was used for gel image analysis, including quantification of spot intensities performed on volume bases (*i.e*. values calculated from the integration of spot optical intensity over spot area).

### MALDI-TOF-MS analysis and protein identification

Samples for MALDI-TOF MS analysis were prepared through the extraction from silver-stained protein spots according to the previous protocol [33]. Enzymatic digestion was performed with 10 mg/ml of sequencing grade modified trypsin (Promega, WI, USA) in 25 nM ammonium bicarbonate (pH 8.0) for overnight at 37°C in a stationary incubator. The trypsin-digested protein spots was analyzed using a MALDI-TOF-MS system (Voyager DE-STR, PE Biosystem, Framingham, MA, U.S.A.) by the Korea Basic Science Institute (Seoul, Republic of Korea), and peptide mass fingerprinting for protein identification was performed using MS-Fit [34]. Spectra were calibrated using a matrix and tryptic autodigestion ion peaks as internal standards. Peptide mass fingerprints were analyzed using the MS-Fit [34]. The identification of a protein with respect to theoretical parameters (pI, molecular mass, etc.) was accepted if the peptide mass matched within a mass tolerance of 10 ppm.

### Bioactivity assay

A hydrolytic enzyme activity of the recombinant cutinase fusion mutant was assessed as described below. The hydrolysis reactions occurred in 96-well microplates at 37°C for 15 min where each well contained 200 μl enzyme/substrate solution comprised of 106.7 μl phosphate buffer (0.1 M, pH 8.0), 13.3 μl Triton X-100 solution (4 g/l), cutinase solution. The reaction was initiated by adding 66.7 μl of substrate reagent solution to each well in the 96-well microplate. Absorbance changes (ΔA_415nm _per min) were measured using a Bio-Rad microplate reader (Tecan, Austria), and solution A with no enzyme solution was used as blank. From the absorbance changes measured at each column, an average absorbance for a specific reaction condition could then be calculated.

## Abbreviations

2-DE, 2-dimensional gel electrophoresis; AID, human activation induced cytidine deaminase; CUT, *Pseudomonas putida *cutinase; EGF, human epidermal growth factor; GAD_448–585, _deletion mutant of human glutamate decarboxylase; G-CSF, human granulocyte colony-stimulating factor; GdnHCl, Guanidine hydrochloride; GST, glutathione-*S*-transferase; hFTN-H, human ferritin heavy chain; hFTN-L, human ferritin light chain; hIL-2, human interleukin-2; MBP, maltose binding protein; mp-INS, minipro-insulin; NACHT, human cold autoinflammatory syndrome 1 protein (NALP3) NACHT domain; PNB, *p*-nitrophenyl butyrate; PNP, *p*-nitrophenyl phamitate; ppGRN, human prepro-ghrelin; Trx, thioredoxin.

## Authors' contributions

JSP, KYH, and JAS carried out all experiments. SHS and KYA carried out 2-dimensional gel electrophoresis and its analysis. JHL and SWK performed HPLC analysis. JSP and SJS drafted and revised the manuscript in collaboration with JL. JL also participated in the planning, design, and coordination of the research. All authors read and approved the final manuscript.
